# Muscular Echovariation as a New Biomarker for the Classification of Soleus Muscle Pathology: A Cross-Sectional Study

**DOI:** 10.3390/diagnostics11101884

**Published:** 2021-10-12

**Authors:** Blanca De-la-Cruz-Torres, Carlos Romero-Morales

**Affiliations:** 1 Department of Physiotherapy, University of Seville, Avicena Street, 41009 Seville, Spain; 2 Faculty of Sport Sciences, Universidad Europea, Villaviciosa de Odón, 28670 Madrid, Spain; carlos.romero@universidadeuropea.es

**Keywords:** ultrasound, soleus injury, diagnosis, echovariation, biomarker

## Abstract

Background: Soleus injury is one of the most common soft tissue tears during sport activities. Current classifications of muscle tears are based on symptoms and tear size and they do not contribute suitable evidence-based treatment protocols. The objective of this study was to analyze the most frequent echotexture findings of patients with soleus muscle injury, located in the central intramuscular tendon (IMT), and healthy people to determine whether they behave differently and to propose an ultrasound (US)-based classification. Methods: eighty-four athletes, who played in sport activities comprising lower limbs. Echotexture characteristics of soleus muscle were reviewed for 84 subjects. They were divided based on the muscle echogenicity in three groups (Injury Type 1 group, Injury type 2 group and healthy group). Echointensity (EI) and Echovariation (EV) were taken in all groups like quantitative US variable. Results. The Injury Type 1 group was identified by a hypoechoic area and characterized by a higher EV; and Injury Type 2 group was identified by a fibrotic area and characterized by a lower EV. The echogenic pattern of healthy people obtained an intermediate value of EV between both injured soleus types. Conclusions. EV may be useful to classify different types of soleus muscle pathology according to the echogenicity pattern. An innovative proposed US-based classification system for soleus tears may be used to guide treatment decisions for patients with central tendon injury of soleus muscle.

## 1. Introduction

Soleus pathologies are one of the most popular soft tissue tears in sports modalities and they are much more common than gastrocnemius pathologies [1,2]. Current studies describe an intramuscular tendon (IMT) and two aponeuroses (lateral and medial) in the soleus muscle [3,4]. Although the IMT may present variability between individuals, it is positioned in the central part of the muscle and has a relevant role like an attaching place of the muscle fibers and being partly of the Achilles tendon [5].

From a topographical point of view, five locations in the soleus muscle were recently identified like possible lesional sites [6]: two myofascial areas (posterior and anterior tears) and three musculotendinous junction points (proximal medial, proximal lateral and distal central tendon tears). Clinically, IMT injury is the most prevalent, being very common in sporting populations, such as dancers or soccer players [5,7]. This injury is characterized by several symptoms that the athlete describes as overload during sports activity, stiffness or lack of elasticity during stretching. The IMT ruptures may create a hypertrophic intramuscular connective tissue scar in the muscle [8]. In addition, they could be considered as non-limiting injuries for sports performance.

Although several physiological parameters have been used as biomarkers in muscle damage [9], their quantification was expensive and time-consuming. Describing a muscle ultrasound biomarker that allows clinicians to diagnose a muscle injury and determine the characteristics of the injury state will be a relevant advance.

Muscle ultrasonography is an easily accessible, low-cost and repeatable tool that provides the image analysis for the classification of muscle tears by clinicians. In 2017, Loizides et al. [10] affirmed that US may be considered as the first-line imaging modality to diagnosis and follow-up of musculoskeletal injuries. The main echotexture parameters are echointensity (EI) and echovariation (EV). The EI is considered as the pixel intensity quantification of an ultrasound image and the EV is determined by the relation between standard deviation (SD) and the EI. Both parameters may express different physiological conditions and their employment has been raised lately in order to obtain a better knowledge of healthy and injured muscle characteristics. Therefore, EI has been used to analyze the muscle quality in healthy populations [11,12] and in patients with degenerative muscle pathologies [13,14,15]. Echointensity changes have been considered as a disruption of normal connective tissue muscle in both degenerative [16,17] and acute conditions [18]. However, the role of the EI parameter as muscle biomarkers in damage status is still limited. For a better interpretation of the muscle echogenicity, EV has been currently employed in combination with EI. To our knowledge, EV has been used in patients with plantar fascia pathology [19], chronic lumbopelvic pain [20], dancers with asymptomatic Achilles tendon [21] and individuals with amyotrophic lateral sclerosis [22]. Regarding muscle injuries in sports people, De-la-Cruz-Torres et al. [7] found that central IMT injury of soleus muscle is characterized by a minor EI. However, they found no differences in EV between healthy subjects and patients with soleus pathology. Authors hypothesize that the analysis of EV in soleus pathology should be more exhaustive and differentiate between types of injuries, characterized by presenting hypoechoic areas or fibrotic areas. Therefore, the main goal of this study was to analyze the most frequent echotexture findings of patients with soleus muscle injury, located in the IMT, and healthy people to determine whether they behave differently and to propose an ultrasound (US)-based classification system for soleus injuries. A possible classification of soleus injuries may contribute suitable evidence-based treatment protocols.

## 2. Materials and Methods

### 2.1. Design

A cross-sectional study was carried out in a sports population according to the Strengthening the Reporting of Observational Studies in Epidemiology Statement (STROBE) recommendations [23].

### 2.2. Ethical Considerations

The study was approved by the Ethics Committee of University Hospital Virgen Macarena-Virgen del Rocio (0663-N-19), respecting the Declaration of Helsinki throughout the study. Before being a participant in this study, all the subjects signed a written informed consent form. 

### 2.3. Sample Size Calculation

G*Power software has been employed for the sample size calculation by the difference between 3 groups for a One-way ANOVA model with a total sample of 12 subjects of a pilot trial with a mean of: Injury Type 1 group = 39.18; Injury Type 2 group = 30.01 and healthy group = 39.19 for the EI variable. Thus, with an effect size of 0.35, an α error of 0.05, a power of 0.80 and a number of 3 groups the total sample determined was 84 subjects.

### 2.4. Participants

**Figure 1:**
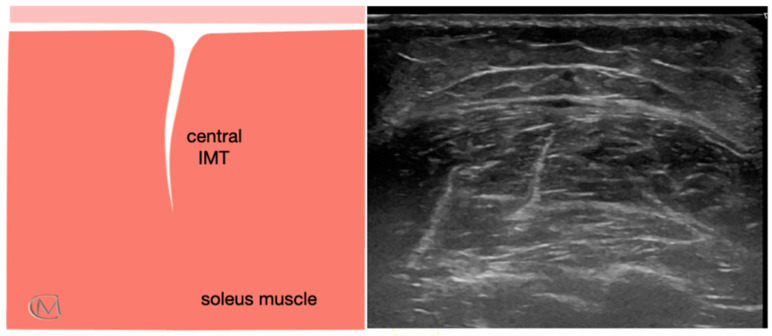
Ultrasound image of soleus muscle in healthy athletes. It shows a normal central IMT. Left panel: anatomic diagram of soleus muscle; Right panel: ultrasound image of the soleus muscle; Abbreviations: IMT, central intramuscular tendon.

**Figure 2:**
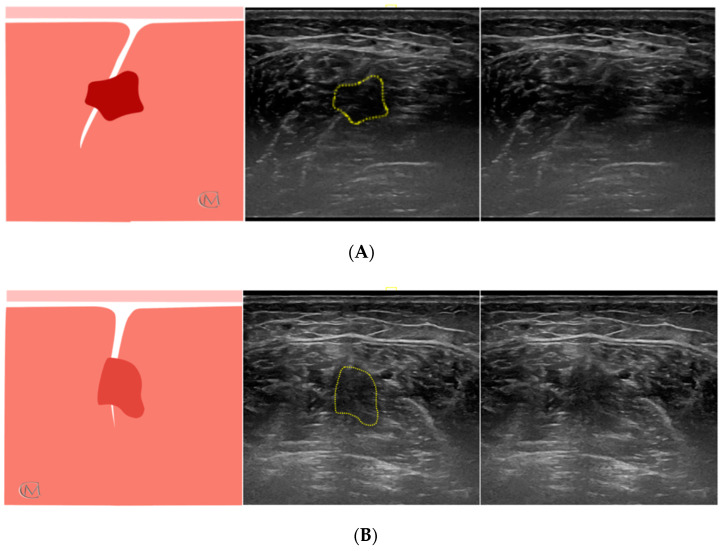
Ultrasound image of soleus muscle in athletes with injured soleus muscle. (**A**) Injury Type 1 group, shows an injured central IMT characterized by hypoechoic imagen; and (**B**) Injury Type 2 group, shows an injured central IMT characterized by fibrotic imagen; Left panel: anatomic diagram of soleus muscle; Middle panel: ultrasound image with marked lesion area (yellow line); Right panel: ultrasound image of the soleus muscle.

Echotexture features of the soleus muscle were reviewed for 84 subjects. They were divided based on the muscle echogenicity in three groups: Injury Type 1 group, healthy group (Figure 1), Injury Type 1 and Injury Type 2 (Figure 2). As in previous studies [7,24], a central IMT tear in the soleus muscle was identified when patients reported having symptoms for at least six months, and they established a self-rated progressive stiffness value greater than or equal to 4 points during sports exercises or during the soleus stretching, measured using the numeric rating scale (NRS) [25]. The enrollment was carried out in a private clinic specialized in sports injuries. First of all, researchers filled out the clinical history of the subjects. The presence of a central IMT injury of the soleus muscle was determined according to the following criteria: clinical history of the individuals, clinical evaluation performed by an experienced therapist and US examination carried out by an US-imaging specialist clinician with more than 15 years of experience.

The selection criteria for the patients were (a) be over 18 years old; (b) athletes who played in sports activities comprising lower limbs (for example, soccer, running, rugby); (b) at least five years practicing the sport; (c) training at least two hours, four days a week; (d) non-acute lower limb pathology at least six months ago; (e) non low back musculoskeletal disorders. Furthermore, inclusion criteria to be included for the healthy group was being a healthy athlete, and for the pathological groups, having a clinical diagnosis of a central IMT injury in the soleus muscle, characterized by muscle stiffness and lack of elasticity when stretching the muscle and corroborated by an experienced clinician.

### 2.5. Clinical Variables

Sociodemographic and clinical variables were registered, such as age, height, weight, body mass index (BMI), sex, dominance side and injured side (Table 1). The pain level at palpation in the injured area and gradual stiffness during the soleus stretching during sport activities were measured using NRS (0 point, no symptoms; 10 points, worse symptoms).

### 2.6. Ultrasonography

All US imaging assessment was performed by two qualified clinicians (over 15 years of experience: B.d.l.C.T and M.C.T), who were blind to the subjects’ muscle status. Each clinician, individually and separately, analyzed the images and then the results were provided to the person who analyzed the data. A high-definition US with a 6–15 MHz linear transducer (S7, General Electrical Healthcare, Chicago, IL, USA) was used to achieve all US images. To the classification of participants, US evaluation was developed in prone position with the feet hanging over the end of the table. Furthermore, a US probe was placed in transverse plane at the central IMT in order to analyze the echogenic model. Ultrasound images were acquired and registered with a depth of 4 cm, 12 MHz of frequency, 51 points of gain, neutral position of the time gain compensation, 2 focus located at 1 and 2 cm each one, respectively, in the lesional area to remain stable during the assessments. The angle of transducer was adapted until the best muscle image was visualized in each image to decrease the existence of artifacts. Researchers captured three images with a resolution of 820 × 614 pixels with 256 gray-scale levels and were saved as .TIFF files without compression or losses.

### 2.7. Image Analysis

Echointensity and echovariation were taken in all groups like quantitative US variables. The imaging study was performed offline with the ImageJ software (Bethesda, MD, USA) by a blinded tester. According to previous study [7], a range of interest (ROI) of 64 × 64 with an 8-bit gray scale using the ROI Manager tool was executed to extract the pixel distribution histogram. The ROI was considered as the muscle area without bone and fascia with the best reflection (Figure 1). Subsequently, echotexture values were captured from the histogram where EI was considered as the mean value of the gray-scale distribution of the pixels, and EV was described as the relation between SD and the mean of pixel distribution (EV = σ/μ × 100), where σ is the SD of the image intensities and μ is the mean value of intensity in each ROI of the three images previously recorded. 

Authors were aware that the muscle echotexture depends on ROI characteristics (for example, the size, shape and location) [26]. To reduce probable mistakes, ROI size was the same in all participants and the location was the one that best identifies the muscle characteristics. Intra-rater reliability in the ROI selection has been reported in a previous study with the same dataset [7] In addition, inter-rater reliability, using the intraclass correlation coefficient, was established in a collection of 15 images as sufficient for clinical evaluation.

### 2.8. Statistics Analysis

SPSS software v.26 (IBM SPSS Statistics; IBM, New York, NY, USA) was used for the statistics. The Kolmogorov-Smirnov test was used to check the normality assumption. A descriptive analysis was carried out for all the subjects separately between groups. One-way ANOVA was employed to assess the homogeneity of the sample and to assess the differences between groups for each variable. In addition, Tukey correction post hoc analyses were developed. Moreover, a multivariate analysis was developed using a linear regression to predict the influence of the descriptive data and group. The dependent variables were EI and EV and the independent variables were group, age, weight, height and BMI. The level of significance was set at *p* < 0.05 with an α error of 0.05 (95% confidence interval) and a desired power of 80% (β error of 0.2).

## 3. Results

Sociodemographic data did not show significant differences (*p* > 0.05) between groups (Table 1). According to Table 2, statistically significant differences (*p* < 0.05) were shown for between-groups comparison for the echotexture variables (EI and EV). Regarding Table 3, post hoc comparison showed statistically significant differences between Injury Type 1 group and Injury Type 2 group, Injury Type 1 group and Healthy group, Healthy group and Injury Type 2 group for EI variable. In addition, EV variable showed significant differences (*p* < 0.05) for Injury Type 1 group and Injury Type 2 group, Injury Type 1 group and Healthy group, Healthy group and Injury Type 2 group. Moreover, inter-reliability values for the EI (ICC = 0.901) and EV (ICC = 0.912) were considered excellent.

According to the linear regression analysis (Table 4), the prediction model for EI (R^2^ = 0.816) was determined by group (absence or presence of plantar fasciitis) and weight. For EV prediction model (R^2^ = 0.243) was determined by group. The rest of the independent variables did not report significant differences between the case and control groups.

## 4. Discussion

The main finding of the present study was to provide a better understanding and new insights about different injured soleus types located in the IMT by ultrasound parameters. In this study, an echotexture classification of injuries affecting the IMT of the soleus muscle is proposed, based on findings in the sports population. The classification may be useful in the clinical setting for the diagnosis, follow up and prevention of musculoskeletal injuries. Specifically, the results show that EV could be a muscle biomarker in athletes with soleus pathology. According to the echogenic pattern, the classification of soleus tears that authors propose is: Injury Type 1, identified by a hypoechoic area and characterized by a higher EV; and Injury Type 2, identified by a fibrotic area and characterized by a lower EV. The echogenic pattern of healthy people should be an intermediate value between both injured soleus types. An intra and inter-rater reliability test was carried out with the objective to increase the reliability of the scores of the assessments. They showed a high reliability in all parameters for this population. 

Considering US as the first-line imaging modality to diagnosis and follow-up of musculoskeletal injuries [10], it was not necessary to perform magnetic resonance imaging like other studies [27]. Nowadays, in the field of sports, there is an urgent need to improve the interpretation of ultrasound images to establish accuracy diagnoses and apply the best treatment options, from a regenerative therapeutic point of view [28,29]. Hence, there is a huge enthusiasm in knowing soleus muscle features and having a better knowledge of pathological characteristics [6,7,30,31]. Based on the special characteristics of IMT tears, these injuries are often not easily recognized, both by therapists and athletes. Indeed, in many scenarios, soleus tears does not require athletes to stop training or playing games [8]. According to our data, authors suggest that the analysis of echotexture variables, such as EV, may be applied as a complementary test for the management of soleus injuries. 

The results described in this research are in line with previous studies that hypothesize the possibility to obtain a muscle biomarker with ultrasonography. In fact, the EV had been analyzed in different muscles of patients with amyotrophic lateral [19] and athletes with chronic lumbopelvic pain [21]. To the best of our knowledge, there are limited studies about the relationship between EV and different classifications of muscle injuries in sport field. Previously, De-la-Cruz-Torres et al. [7] compared the EV of patients with IMT injury of soleus muscle and healthy people, but they found no differences. Authors suggest that this could be because it is needed to determine the injury status to determine its echogenic pattern. Pathologies with the same symptomatology and lesional mechanism may be characterized by a different histology and, therefore, the diagnosis and treatment may be different. Authors hypothesize that IMT tears of the soleus muscle can be considered (Figure 2, Table 3): on the one hand, a re-injury characterized by a hypoechoic image, compatible with edema, and a higher EV (Injury type 1); and on the other hand, a fibrotic injury characterized by a fibrotic image, compatible with fibrosis, and a lower EV (Injury type 2). In this line, future studies are also needed to better explain the characteristics of the pathological features on injured muscle. Knowledge of basic anatomic and histology in combination with pathology imaging are required.

### 4.1. Clinical Applications

The most relevant clinical implication of this study was the proposal of the EV as a muscle biomarker for the classification of soleus injured muscle, based on echogenic patterns. Hence, EV may be used in future studies for the prevention as well as for the management and monitoring of muscle tears. This muscle biomarker may even determine the most effective treatment options for the recovery process. More research on treatment options are needed. It is crucial to highlight that the proposed US-based classification is complementary to other muscle injury classifications.

### 4.2. Limitations and Future Lines

The present study had several limitations. In this study, only ultrasound was used as a diagnostic tool. Comparing different diagnostic strategies for muscle injuries, such as magnetic resonance imaging or laboratory tests, could help to better understand the pathological characteristics of muscle injuries. Furthermore, to establish the role of EV as a diagnostic or follow-up biomarker, this echotexture analysis must be applied throughout the entire recovery process and after applying the treatments. Furthermore, future research may be considered in other muscle and soft tissues disturbances.

## 5. Conclusions

Authors suggest that EV may be useful to classify different types of soleus muscle pathology according to the echogenicity pattern. An innovative proposed US-based classification system for soleus tears may be used to guide treatment decisions for patients with central tendon injury of soleus muscle. The proposed classification may be easily performed in the clinical setting with the aim to improve the diagnosis, management and monitoring their return to sport after soleus muscle injury.

## Figures and Tables

**Table 1:** Sociodemographic data of the sample.

Data	Total Sample (*n* = 84)	Injury Type 1 Group (*n* = 28)	Injury Type 2 Group (*n* = 28)	Healthy Group (*n* = 28)	*p* Value
Age, y	26.86 ± 8.85	25.57 ± 7.23	26.28 ± 10.62	28.75 ± 8.36	0.375
Weight, kg	68.71 ± 10.47	68.81 ± 12.84	65.14 ± 8.41	64.39 ± 9.87	0.395
Height, m	1.69 ± 0.08	1.70 ± 0.10	1.67 ± 0.62	1.71 ± 0.87	0.283
BMI, kg/m^2^	23.46 ± 3.00	23.82 ± 3.18	23.38 ± 3.88	23.19 ± 1.94	0.745
Gender (F/M)	45/39	16/12	15/13	14/14	-
Dominance side (R/L)	76/8	25/3	26/2	25/3	-
Injured side (R/L)	42/32	17/11	16/12	19/9	-

Abbreviations: F, female; M, male; L, left; R, right.

**Table 2:** One-way ANOVA for the Numerical Rating Scale (NRS) during sport activity and at palpation in soleus injury, Echointensity (EI), and Echovariation (EV) variables.

Data	Injury Type 1 Group	Injury Type 2 Group	Healthy Group	*p* Value
NRS during activity (points)	5.39 ± 1.64	5.89 ± 1.49	N/A	N/A
NRS at palpation (points)	5.33 ± 1.34	6.03 ± 1.34	N/A	N/A
Echointensity (EI)	19.64 ± 7.38	48.62 ± 8.83	64.53 ± 10.51	(178.8) 0.001
Echovariation (EV)	53.21 ± 19.23	22.54 ± 9.08	32.93 ± 7.36	(40.34) 0.001

**Table 3:** Bonferroni correction for Numerical Rating Scale (NRS) during sport activity and at palpation in soleus injury, Echointensity (EI), and Echovariation (EV) variables.

Data	Group	Group	Mean Difference (95% CI Minimum–Maximum)	*p* Value
NRS during activity (points)				
	Injury Type 1	Injury Type 2	−0.500 (−1.35–0.28)	0.316
	Injury Type 1	Healthy	5.392 (4.61–6.25)	0.001
	Healthy	Injury Type 2	−5.89 (5.14–6.78)	0.001
NRS at palpation (points)				
	Injury Type 1	Injury Type 2	−0.696 (−1.24–0.14)	0.052
	Injury Type 1	Healthy	5.339 (5.23–6.62)	0.001
	Healthy	Injury Type 2	−6.035 (−6.07–−4.68)	0.001
Echointensity (EI)				
	Injury Type 1	Injury Type 2	−28.976 (−34.72––−23.11)	0.001
	Injury Type 1	Healthy	−44.887 (−51.90–−40.30)	0.001
	Healthy	Injury Type 2	15.911 (11.38–22.99)	0.001
Echovariation (EV)				
	Injury Type 1	Injury Type 2	30.673 (22.63–43.44)	0.001
	Injury Type 1	Healthy	20.279 (13.62–34.44)	0.001
	Healthy	Injury Type 2	10.393 (−1.39–19.41)	0.010

**Table 4:** Multivariate predictive analysis for EI and EV variables for patients with plantar fasciitis and controls.

Parameter	Model	Beta Coefficient	Model *R*^2^
EI	96.914		0.816
	22.561 * Group	0.874 ‡	
	−59.737 * Weight (kg)	−0.249 ‡	
EV	7.371		
	−12.173 * Group	−0.464 ‡	0.243

Abbreviations: EI, echointensity; EV, echovariation. * Multiplay: Group (control = 0; Plantar fasciitis = 1); ‡ *p*-value < 0.001 for a 95% confidence interval was shown.

## Data Availability

Data sharing is not applicable to this article.
